# Prevalence of Familial Hypercholesterolemia and Its Association with Cardiovascular Risk in a Cross-Sectional Adult Population

**DOI:** 10.3390/jcm14228213

**Published:** 2025-11-19

**Authors:** Kairat Davletov, Indira Baibolsynova, Nurdaulet Umirbekov, Ainagul Auyelbekova, Sergey Lee, Ruslan Kulmanbetov, Mukhtar Kulimbet

**Affiliations:** 1 Scientific Technologic Park, Asfendiyarov Kazakh National Medical University, Almaty 050012, Kazakhstan; davletovkairat@gmail.com; 2 Science Department, Asfendiyarov Kazakh National Medical University, Almaty 050012, Kazakhstan; baibolsynova.i@kaznmu.kz (I.B.); lee.s@kaznmu.kz (S.L.); 3 City Polyclinic №32, Almaty 050054, Kazakhstan; sbaimbetov32@gmail.com (N.U.); ainagul_32@mail.ru (A.A.); 4 Department of Orthodontics, Asfendiyarov Kazakh National Medical University, Almaty 050012, Kazakhstan

**Keywords:** familial hypercholesterolemia, low-density lipoprotein cholesterol, cardiovascular disease, Kazakhstan

## Abstract

**Background/Objectives**: Familial hypercholesterolemia (FH) is an inherited lipid disorder that markedly elevates lifetime risk of premature cardiovascular disease (CVD), yet remains under-recognized globally. We aimed to estimate the prevalence of phenotype-defined FH among adults in Almaty, Kazakhstan, describe lipid profiles and treatment patterns, and examine the association between FH status and prevalent CVD. **Methods**: We conducted a cross-sectional analysis of routinely collected clinical and laboratory data from adults (≥18 years) recorded between March 2023 and March 2024. FH status was classified using Dutch Lipid Clinic Network criteria (probable: 6–8 points; possible: 3–5). Outcomes included lipid measures, statin use, and CVD. Group comparisons used standard tests; multivariable logistic regression estimated adjusted odds ratios (AORs) for prevalent CVD, controlling for age, sex, smoking, body mass index (BMI), systolic blood pressure (SBP), and LDL-C. **Results**: Among 2468 participants (mean age 45.2 ± 14.3 years; 64.7% women), FH prevalence was 0.4% probable (*n* = 10) and 6.7% possible (*n* = 166). FH groups had substantially higher LDL-C and higher systolic blood pressure. Overall statin use was 6.7%; within FH, 13.3% (possible) and 10.0% (probable) used statins. Prevalent CVD affected 18.6% overall, rising to 48.2% (possible FH) and 60.0% (probable FH) (*p* < 0.001). After adjustment, FH remained independently associated with CVD (AOR 8.15, 95%CI 5.30–12.53 for possible; AOR 40.60, 95%CI 9.15–180.2 for probable vs. non-FH). Age and BMI were positively associated with CVD; LDL-C showed an inverse association consistent with treatment confounding. **Conclusions**: In this Kazakhstani adult cohort, phenotype-defined FH was present in 0.4% probable and 6.7% possible with a high burden of prevalent CVD and low statin use. These findings highlight substantial missed opportunities for early detection and aggressive lipid-lowering therapy, and support implementing FH identification, treatment intensification, and family-based cascade strategies in Kazakhstan.

## 1. Introduction

Familial hypercholesterolemia (FH) is a common inherited disorder of lipid metabolism characterized by lifelong elevated low-density lipoprotein cholesterol (LDL-C) levels and a significantly increased risk of premature cardiovascular disease (CVD) [1]. Estimates of FH prevalence are approximately 0.26–0.39% of the general population (roughly 1 in 313 individuals) worldwide [2,3], making it one of the most prevalent genetic conditions.

There is some geographic and ethnic variation in reported prevalence [4,5]. These differences may reflect founder effects, population demographics, or varying diagnostic criteria. Nevertheless, across regions the prevalence of FH in those with established heart disease is dramatically higher [3]. This highlights the substantial contribution of undiagnosed FH to premature atherosclerosis globally.

Despite its frequency and clinical significance, most individuals with FH remain unidentified and untreated. It is estimated that less than 1% of FH cases are currently recognized in most countries [6]. Moreover, even among those diagnosed with FH, many do not receive adequate therapy to achieve recommended LDL-C targets [7]. The reasons include lack of awareness, insufficient screening, and limited access to potent lipid-lowering therapies in some regions [8]. Early diagnosis and aggressive treatment of FH (with high-intensity statins, ezetimibe, PCSK9 inhibitors) can dramatically improve outcomes. Dietary and lifestyle measures alone are usually insufficient to counteract the extreme LDL elevation in FH. Therefore, identifying individuals with FH and instituting therapy early is a public health priority.

To date, there is a notable gap in the epidemiological data on FH in Central Asia. Most prevalence studies have been conducted in Europe, North America, East Asia, and Australia, whereas regions such as South/Central Asia and Africa are under-studied [6]. Kazakhstan, a large Central Asian nation, has lacked published data on the burden of FH.

The present study aims to address this gap by examining the prevalence of FH in Almaty region among adults in Kazakhstan and evaluating their lipid profiles, cardiovascular comorbidities, and treatment patterns. Understanding the prevalence, clinical characteristics and treatment patterns of FH in Kazakhstan is crucial for informing national guidelines, designing cascade testing programs and allocating resources for lipid-lowering therapies.

## 2. Materials and Methods

### 2.1. The Study Design

This was a cross-sectional study of data obtained from patient records at City policlinic №32 Almaty, Kazakhstan. We analyzed existing clinical and laboratory data recorded between March 2023 and March 2024. The dataset primarily consisted of routine health check-up records and was supplemented by targeted telephone interviews for clarification of missing or unclear variables. This approach ensured a more complete dataset while maintaining the retrospective nature of this study. The study design and reporting followed the STROBE guidelines for observational research to ensure methodological rigor and transparency in analysis.

### 2.2. Study Population

**Figure 1:**
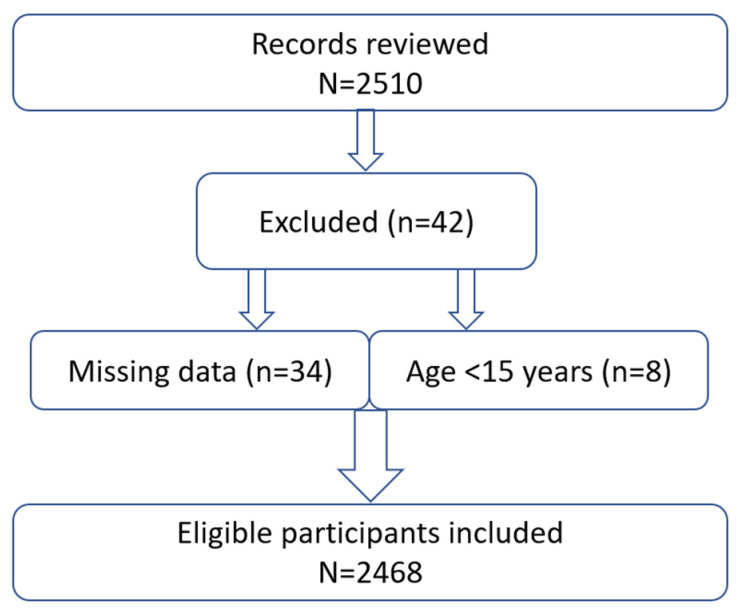
Participants’ inclusion flowchart.

We included all patients aged ≥18 years who had a documented fasting lipid profile and relevant clinical data in the source records during the study period. A total of *N* = 2468 participants met these criteria and were included in the final analysis. Figure 1 illustrates the inclusion process. Patients below 18 years of age or those lacking essential data (such as absence of any lipid measurement) were excluded; however, due to the comprehensive data retrieval efforts, missing data were minimal. To ensure transparency and reproducibility, we examined the extent of missing data across all study variables. Less than 5% of values were missing for any covariate or outcome, and missingness appeared to be unrelated to FH status or cardiovascular outcomes. In line with recommendations stating that complete case analysis is appropriate when the proportion of missing data is very minimal and unlikely to introduce systematic bias, our primary analyses were limited to participants with complete data. The sample comprised individuals from both urban and rural clinics in Kazakhstan, with diverse ethnic backgrounds (e.g., Kazakh, Russian, and other ethnicities represented). Basic demographic information (age, sex, ethnicity), clinical history, and laboratory results were collected from the records at baseline.

### 2.3. Sample Size and Recruitment

No formal sample size calculation was performed prior to this study since all eligible records within the specified timeframe were included. We recognized that sample size is critical for the validity of study conclusions—using an inadequate sample can lead to inconclusive or misleading results. To assess the sufficiency of our sample, we conducted a post hoc power analysis. Using the observed rates of cardiovascular disease in the FH group versus non-FH group, we found that the achieved sample of 2468 provided excellent power (>95%) to detect a clinically meaningful difference at α = 0.05. For instance, probable/possible FH patients had a markedly higher prevalence of CVD than those without FH (approximately 52% vs. 16%, respectively), a difference which our sample could detect with >99% power. Even for more modest differences—such as the higher statin use in FH patients (around 13%) compared to others (6%)—the power exceeded 90%. These calculations indicate that our study was well-powered for the main comparisons of interest. In other words, the risk of Type II error (falsely missing a true effect due to insufficient sample) was very low given our sample size and the effect sizes observed. All power calculations were performed using the R statistical software (version 4.3.0) with the pwr package, cross-validated by the ClinCalc online power calculator.

### 2.4. Variables and Definitions

The primary outcome of interest was the presence of familial hypercholesterolemia (FH) phenotype. We classified each participant’s FH status using the Dutch Lipid Clinic Network (DLCN) criteria [9]. The DLCN is based on family history of premature cardiovascular disease (CVD), personal history of CVD, untreated low-density lipoprotein cholesterol (LDL-C) levels, and physical stigmata (e.g., tendon xanthomas). According to these criteria, a DLCN score of 6–8 indicates “probable FH” and 3–5 indicates “possible FH”, while a score < 3 makes FH unlikely. In our dataset, no individuals had genetic testing or tendon xanthomas documented, so none met criteria for “definite FH” (which requires score > 8 or a pathogenic mutation). We therefore focused on probable FH cases (DLCN ≥ 6) versus others for analysis, and also noted those with possible FH. A binary variable FH probable was defined as “yes” for participants meeting probable FH criteria and “no” otherwise.

We extracted laboratory values including total cholesterol, LDL-C, high-density lipoprotein cholesterol (HDL-C), triglycerides, serum creatinine, and glycated hemoglobin (HbA1c) from the records. All blood samples were collected after an overnight fast and analyzed in accredited laboratories using standard enzymatic methods (values reported in SI units, mmol/L for lipids and % for HbA1c). Anthropometrics (height in cm, weight in kg) were recorded and used to calculate body mass index (BMI, kg/m^2^). In cases where height or weight was missing, patients provided the information during telephone follow-up. We used LDL-C values recorded in the electronic medical records at the time of clinical evaluation. For participants not yet receiving lipid-lowering therapy, these values represent untreated LDL-C. For those already on statins or other lipid-lowering drugs, we used the highest documented LDL-C level measured before or at initiation of therapy; if such data were unavailable, the on-treatment LDL-C was analyzed. This approach aligns with contemporary guidelines [10], which recommend that familial hypercholesterolemia criteria be based on untreated LDL-C and, for treated patients, that clinicians refer to the LDL-C level that originally triggered therapy. We acknowledge that reliance on on-treatment LDL-C may lead to underestimation of FH severity.

We obtained data on sociodemographic factors (education level in years and categories, marital status, occupation) and lifestyle behaviors. Smoking status and alcohol intake were recorded (categorized as current, former, or never). We also noted whether each patient had a documented history of cardiovascular disease (CVD), defined as a prior diagnosis of coronary heart disease, myocardial infarction, stroke, or peripheral arterial disease (as recorded in their medical chart). Family history of CVD and hypertension in first-degree relatives was captured as binary variables (relative MI for myocardial infarction and relative hypertension). In cases where some information was missing, patients provided the information during telephone follow-up. The follow-up calls were conducted by trained nurses who received standardized training and used a structured script. The script included an introduction explaining this study, confirmation of eligibility, collection of missing variables. Participants were contacted three times on different days and at different times. The verbal informed consent was obtained at the start of each call, and participants were assured that their responses would remain confidential. The responses obtained from telephone interviews were cross-checked against electronic medical records (EMRs) or paper charts to verify diagnoses, LDL-C levels, and prescription histories. Discrepancies were resolved by consulting the attending clinicians or by reviewing laboratory reports. Medication use was recorded, specifically current statin therapy and aspirin use (yes/no). Main variables and their definitions are summarized in Table 1.

### 2.5. Ethics

This study was conducted in accordance with the Declaration of Helsinki, and approved by the Local Ethics Committee of Asfendiyarov Kazakh National Medical University, Almaty, Republic of Kazakhstan (protocol code: 11(134); date of approval: 4 November 2022).

### 2.6. Statistical Analysis

We used SAS OnDemand for Academics (release 3.81, Carry, NC, USA) and R (v4.3.0) for data analysis. Prior to analysis, data were checked for completeness and accuracy. Continuous variables were summarized as mean ± standard deviation (SD) if approximately normally distributed, or median with interquartile range (IQR) if skewed. Categorical variables were summarized as frequencies and percentages. For continuous variables (e.g., age, LDL-C levels), group comparisons were made using the independent samples *t*-test if distributions were normal (verified by Shapiro–Wilk test) or the Mann–Whitney U test for non-normal data. For categorical variables, we used the chi-square test (χ^2^) or Fisher’s exact test (if any expected cell count < 5) to assess differences in proportions. To quantify associations and adjust for potential confounders, we performed multivariable logistic regression analyses. Results of regression analyses are reported as odds ratios (OR) with 95% confidence intervals (CI). We checked model assumptions and the goodness-of-fit (using Hosmer–Lemeshow test). A two-tailed *p*-value < 0.05 was considered statistically significant for all analyses. Statistical analysis and reporting were conducted in accordance with STROBE recommendations and epidemiologic standards.

## 3. Results

According to Table 2, a total of 2468 participants were included in this study, with a mean age of 45.2 ± 14.3 years. The study population was predominantly composed of middle-aged (30–49 years—41.0%), educated (60.1% holding graduate and 2.9% postgraduate degrees), and employed women (64.7%) of Kazakh ethnicity (63.3%). The estimated prevalence of possible and probable FH was 6.7% and 0.4%, respectively, while CVD affected nearly one-fifth of participants (18.6%). Despite a substantial proportion exhibiting lipid abnormalities and cardiovascular risk factors, statin use remained low (6.7%). Mean lipid and metabolic profiles indicated borderline dyslipidemia, with average LDL-C above optimal levels.

According to Table 3, participants with possible/probable FH were generally older and had markedly higher lipid levels than those without FH. The mean age of individuals without FH was 44.5 years, compared to 55.2 years in the possible FH group and 51.0 years in the probable FH group (*p* < 0.001). FH groups did not differ significantly in sex ratio: both FH and non-FH groups were about two-thirds female. Systolic blood pressure was higher in the FH groups: the mean systolic blood pressure was 122.9 ± 18.2 mmHg in non-FH, 128.2 ± 18.1 in possible FH, and 134.7 ± 21.4 in probable FH (*p* < 0.001). Body mass index was also significantly different across categories (25.9 in non-FH, 27.1 in possible FH, 25.4 in probable FH; *p* < 0.001). There were no significant differences in glycemic status by FH category. Only 10% of probable FH and 13% of possible FH participants use statin therapy. The prevalence of CVD among possible and probable FH groups were 48.2% and 60% accordingly (*p* < 0.001).

**Figure 2:**
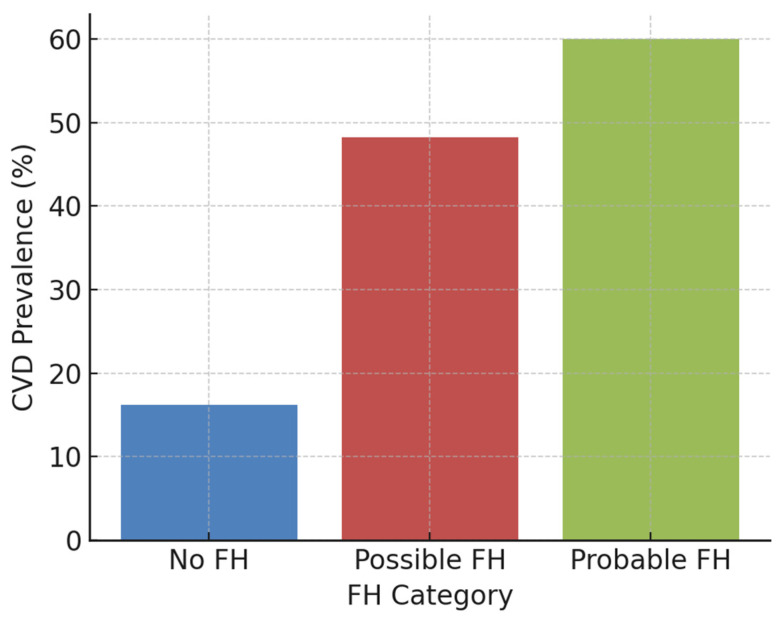
A bar chart showing cardiovascular disease prevalence across FH categories (No FH, Possible FH, Probable FH).

Figure 2 shows the CVD prevalence across FH categories. Notably, nearly two-thirds of the probable FH group had experienced a cardiovascular event.

Table 4 shows whether FH status predicts CVD independent of other risk factors using multivariable logistic regression. After controlling for age, sex, smoking status, body mass index, SBP, and LDL-C level, FH status remained a significant independent predictor of CVD. Participants with possible FH had an adjusted OR (AOR) of 8.15 (95% confidence interval 5.30–12.53; *p* < 0.001) for having CVD compared to those without FH. Those with probable FH had an even higher AOR of 40.6 (95% CI 9.15–180.2; *p* < 0.001 vs. non-FH). Increasing age, as expected, was also a strong predictor of CVD (AOR 1.035 per year, 95%CI 1.025–1.044). Interestingly, male sex was negatively associated with CVD in our sample (AOR 0.71, 95%CI 0.50–0.90 for male vs. female, *p* = 0.005). Smoking did not show significant associations with CVD in the multivariable model. Higher body mass index was modestly associated with CVD (AOR 1.04 per kg/m^2^, *p* = 0.001). Notably, LDL-C (modeled as a continuous variable) was inversely associated with prevalent CVD after adjustment (AOR 0.61 per mmol/L increase, *p* < 0.001).

## 4. Discussion

### 4.1. Main Findings and Their Explanations

In this cross-sectional study of a Kazakhstani adult population, using Dutch Lipid Clinic Network (DLCN) criteria, we observed that those with probable and possible FH had markedly elevated LDL-C levels and a very high burden of cardiovascular disease—nearly half had experienced a CVD event. Furthermore, FH status was a strong independent predictor of having CVD, even after controlling for LDL levels and other risk factors, consistent with the notion that lifelong exposure to high cholesterol confers excess risk. Despite their high risk, the majority of individuals meeting FH criteria were not receiving statin therapy. To our knowledge, this study is among the first to document the epidemiology of FH and its impact on CVD in Kazakhstani population, revealing both a substantial hidden burden of FH and missed opportunities for prevention.

The prevalence of probable FH in our study is nearly consistent with global statistics [3]. Large studies in the US [11] and Europe [12] have converged on roughly 0.4% prevalence of FH using genetic or clinical criteria. The prevalence of possible FH in our study is higher compared to other countries [13,14], which can be explained by several factors. First, our cohort has a somewhat older age distribution, and prevalence of FH rises with age as cholesterol accumulates and clinical events accrue. Second, there may be regional or lifestyle influences: diets rich in animal fat and low use of preventive medication in our setting might lead to more people having LDL levels above the diagnostic thresholds. The possible FH group is still important clinically because it flags people with significant hypercholesterolemia who may benefit from aggressive prevention.

We found that individuals meeting FH criteria had 7-fold higher odds of CVD compared to others, even after controlling for LDL levels and adjusting for major risk factors. The similar results were seen in other pooled cohort analysis [15], where findings indicate that genetic FH increases risk beyond the effect of the LDL measurement alone. The excess risk is thought to arise from the cumulative exposure to high LDL from a young age and perhaps other metabolic factors that accompany the FH genotype [15]. Our results are consistent with this, even when we adjust for a single LDL measurement, FH individuals had far more CVD, underscoring the importance of lifelong cholesterol burden.

An interesting finding in our study was the low rate of statin therapy among FH individuals. Only 13% were on treatment, and essentially none of the FH subjects without CVD were being treated preventively. This reflects either lack of diagnosis or lack of initiation of therapy even if recognized. It is consistent with global observations that FH is under-treated—for instance, an analysis in the US found fewer than 50% of FH patients were on any lipid-lowering medication and even fewer on high-intensity statins [16,17]. In our cohort, the situation appears even more concerning. The fact that FH status lost significance as a predictor of statin use after accounting for CVD suggests that many patients only received therapy after a coronary event. Ideally, FH should be managed with early and aggressive primary prevention, meaning high-dose statins (often combined with ezetimibe or PCSK9 inhibitors) started as soon as the condition is identified, often in young adulthood or even childhood [18]. In addition, PCSK9 inhibitors have a pleotropic effect such as atherosclerotic plaque stability, endothelial function, microcirculation, inflammation, and platelet reactivity along with the intensive lipid lowering effect as noted from recent reviews [19]. However, according to our data, early intervention is not happening in this population. This may be due to limited awareness of FH among healthcare providers, cost or availability issues with medications, or patients discontinuing therapy (possibly influenced by misconceptions about statins). The net result is that many FH individuals remain exposed to extremely high lifelong LDL levels, resulting in preventable heart attacks. As highlighted by Alonso et al. (2020) in a review on barriers to FH care, challenges include cost and access to medications, poor long-term adherence, limited familiarity with FH among general practitioners and low perception of cardiovascular risk [20]. Qualitative evidence syntheses and registry studies further show that negative perceptions of medication, beliefs that treatment is unnecessary when patients feel asymptomatic, concerns about side-effects and lack of knowledge about treatment or disease are significant barriers to adherence [21].

### 4.2. Future Implications and Directions

Our findings reveal a profound primary-prevention gap in FH care. In our cohort, only 13% of FH individuals were on statin therapy—and none without established CVD were treated—despite lifelong exposure to very high LDL-C. Importantly, the loss of statistical association between FH status and statin use after adjusting for CVD in our data suggests treatment is frequently initiated only after an event (secondary prevention), rather than at diagnosis (primary prevention). Improving FH management will likely require concerted efforts in clinician education, patient awareness, and health system policies. For our region, establishing an FH registry and patient network could help track diagnosis rates and facilitate cascade screening. Also, integrating automatic alerts in laboratory systems (for example, a flag when LDL is above a certain threshold) can prompt physicians to evaluate for FH. Family physicians and cardiologists should be trained to recognize physical stigmata of FH (like tendon xanthomas, corneal arcus in young patients) and to take detailed family histories of cholesterol and premature CVD.

Experiences from Asian and Eastern European FH registries provide clear lessons on how health systems can strengthen early FH detection. Large regional analyses show that systematic approaches (population LDL screening, electronic alerts, and structured care pathways) significantly increase identification rates across Japan, Korea, and China [22,23]. In Poland, national FH cascade testing programs—integrated with the health insurance system and laboratory networks—have dramatically increased the number of diagnosed index cases and relatives. The Czech Republic’s long-standing genetic FH registry demonstrates that combining genetic testing with clinical criteria can substantially reduce misclassification and guide more cost-effective use of lipid-lowering therapy [23].

Furthermore, our data highlight how phenotypic criteria tend to overestimate FH prevalence relative to genetic prevalence. This pattern has been reported in other studies as well. For example, in the US NHANES dataset, the prevalence of “clinical FH” by cholesterol cutoffs was higher than the mutation-confirmed prevalence [2]. What our findings add is a context from a country where background cholesterol levels are relatively high and preventive treatment is low—under these conditions, the clinical FH criteria label a surprisingly large segment of adults. This suggests that, in such settings, relying solely on clinical criteria without genetic confirmation could overwhelm healthcare services with “possible FH” cases, many of whom may actually have polygenic hypercholesterolemia. An implication is that genetic testing or more specific criteria might be needed to distinguish true FH in these populations. Nevertheless, even those with phenotypic “false positives” have markedly elevated LDL and deserve attention for cardiovascular prevention.

Future research in this area should incorporate genetic testing to precisely determine FH prevalence and to study genotype-phenotype correlations in this population. It would be valuable to know, for instance, which LDLR or other mutations are common in such populations. A longitudinal cohort study is also warranted to follow individuals with high LDL/FH and see their actual CVD event rates and how interventions change outcomes. Such data could persuade health authorities about the cost-effectiveness of investing in FH detection. Additionally, qualitative research might explore why treatment rates are low—do patients decline therapy due to side effect fears or do doctors under-prescribe. Understanding these barriers would help tailor public health messages.

### 4.3. Limitations

Several limitations of our study should be acknowledged. First, the diagnosis of FH was based on clinical scoring (DLCN criteria) without genetic testing. As discussed, this can lead to misclassification—some individuals labeled “FH” might not carry a causative mutation, while a few with mutations could have been missed if their phenotype was mild. Thus, our prevalence of “FH” must be interpreted as possible or probable FH by clinical criteria rather than confirmed genetic FH. In addition, the DLCN criteria capture clinical features but do not definitively distinguish between monogenic FH and polygenic or environmentally driven hypercholesterolemia especially for individuals categorized as possible FH. Therefore, misclassification among the possible FH group likely biases results toward the null, in other words the true association between genetically confirmed FH and ASCVD risk is expected to be stronger than what we could observe using only clinical FH definitions.

Second, this study is cross-sectional which means we cannot establish temporality or causation. For example, we used current LDL levels in the regression models, but many CVD cases were likely on treatment (lowering their LDL), which complicated interpretation of LDL’s effect. Our finding of an inverse relationship between LDL level and statin use and CVD presence is evidence of this treatment-confounding—those with CVD often had lower LDL (due to therapy) than those without CVD. A longitudinal study would be needed to properly assess how baseline LDL and FH status predict future events. In addition, a sensitivity analysis excluding individuals on lipid-lowering therapy could reduce treatment-related distortion of LDL-C, but it would introduce selection bias since treated individuals are typically at higher baseline risk. Although correction factors are sometimes used to estimate pre-treatment LDL-C, this requires detailed data on statin type, dose, adherence, and duration, which were partially unavailable in our dataset. Due to these methodological and data constraints, treatment-corrected LDL-C was not applied, though such analyses would strengthen causal interpretation in future studies.

Third, the probable FH subgroup was very small (*n* = 10), which means any results pertaining to that group (like the exact OR for CVD or statin use) should be interpreted with caution due to wide confidence intervals. We combined possible + probable for some analyses to improve power.

Fourth, our sample, while population-based, might not be perfectly representative of the entire country or region. The proportion of women was high (60%) compared to national estimates. This could affect generalizability of absolute prevalence numbers. However, the internal comparisons (FH vs. non-FH differences) should be robust. Since our primary objective was to characterize FH burden in the available real-world sample rather than produce nationally representative prevalence estimates we did not apply the weighting or stratified analysis. Future population-based surveys with balanced sampling frames—or post-stratification weighting—will be essential to improve representativeness.

Fifth, we did not have data on some factors that could be relevant—for example, lipoprotein(a) levels, which are often elevated in FH and independently contribute to risk, were not measured [24]. In addition, lack of socioeconomic or dietary data which could influence lipid levels were not measure.

Lastly, recall bias could influence the results since some variables such as family history was self-reported.

## 5. Conclusions

In summary, our study provides the first detailed look at FH in Kazakhstani population and reveals a high prevalence of phenotype-defined FH with corresponding high cardiovascular risk. Most affected individuals are not receiving life-saving therapies. Our results underscore that familial hypercholesterolemia is a significant and under-addressed public health issue in Kazakhstan. Improving the identification of FH through routine cholesterol screening and clinical criteria and promptly initiating aggressive lipid-lowering treatment for those affected should be priorities to reduce premature cardiovascular events.

## Figures and Tables

**Table 1:** Variables and their definitions and measurements.

Variable Name	Definitions/Measurements
Familial Hypercholesterolemia (FH)	Classified by Dutch Lipid Clinic Network score: “probable FH” if score 6–8, “possible FH” if 3–5, “unlikely” if <3.
LDL-C, HDL-C, Total Cholesterol, Triglycerides	Fasting lipid profile (mmol/L) measured via standard enzymatic assays in certified labs.
HbA1c	Glycated hemoglobin (%), measured by high-performance liquid chromatography (NGSP/DCCT-aligned).
Body Mass Index (BMI)	Calculated as weight(kg)/[height(m)]^2^ from medical records.
Cardiovascular Disease (CVD)	History of coronary heart disease, myocardial infarction, stroke, or peripheral arterial disease (from medical records).
Family History	First-degree relative with hypertension or premature CVD (men <55 or women <60 years), recorded as yes/no for each.
Smoking Status	Categorized as current smoker, former smoker, or never smoker.
Alcohol Intake	Consumption of alcohol (yes/no).
Statin Use	Currently on statin medication (yes/no) as recorded in prescription list.
Aspirin Use	Currently on aspirin therapy (yes/no) as recorded in prescription list.

**Table 2:** Demographic characteristics.

Characteristics	*N* (2468)	Percent (%)
Gender		
female	1596	64.67
male	872	35.33
Age, years	2468	45.2 (14.3)
Familial hypercholesterolemia status		
no	2292	92.87
possible	166	6.73
probable	10	0.41
Education level		
graduate	1482	60.05
postgraduate	72	2.92
school completed (11th grade)	789	31.97
school completed (9th grade)	125	5.06
Ethnicity		
Kazakh	1563	63.33
Russian	566	22.93
other	339	13.74
Occupation		
private worker	1215	49.23
public worker	422	17.1
retiree	378	15.32
student	105	4.25
unemployed	348	14.1
Smoking status		
no	2081	84.32
yes	387	15.68
Alcohol intake		
no	1052	42.63
yes	1416	57.37
CVD		
no	2010	81.44
yes	458	18.56
Statin use		
no	2302	93.27
yes	166	6.73
Hypertension among relatives		
do not know	36	1.46
no	1526	61.83
yes	906	36.71
MI among relatives		
do not know	31	1.26
no	2101	85.13
yes	336	13.61
HDL-C, mmol/L	2468	1.37 (0.32)
LDL-C, mmol/L	2468	3.34 (0.94)
Triglyceride, mmol/L	2468	1.36 (1.06)
Total cholesterol, mmol/L	2468	5.24 (1.32)
HbA1c, %	2468	5.57 (0.93)
SBP, mmHg	2468	124.01 (18.54)
DBP, mmHg	2468	81.69 (11.01)
Body mass index	2468	25.92 (4.75)

Values are presented as no (%), mean ± SD (standard deviation) and median (min–max); HDL-C, high-density lipoprotein; LDL-C, Low-density lipoprotein cholesterol; HbA1c, hemoglobin A1C; MI, myocardial infarction; CVD, cardiovascular disease; SBP, systolic blood pressure; DBP, diastolic blood pressure.

**Table 3:** Participant characteristics stratified by FH status.

Characteristic	No FH (*n* = 2292)	Possible FH (*n* = 166)	Probable FH (*n* = 10)	*p*-Value
Age, years	44.5 ± 14.3	55.2 ± 10.0	51.0 ± 9.7	<0.001
Female, %	64.5%	67.5%	50.0%	0.2834
LDL-C, mmol/L	3.20 ± 0.80	5.04 ± 0.63	6.74 ± 1.07	<0.001
HDL-C, mmol/L	1.36 ± 0.39	1.53 ± 0.40	1.80 ± 0.47	<0.001
Triglycerides, mmol/L	1.29 ± 0.77	2.03 ± 1.01	1.72 ± 0.58	<0.001
Total cholesterol, mmol/L	5.06 ± 1.01	7.40 ± 1.07	9.70 ± 1.35	<0.001
HbA1c, %	5.56 ± 0.87	5.67 ± 0.74	5.70 ± 0.64	0.3644
BMI, kg/m^2^	25.9 ± 4.7	27.1 ± 5.1	25.4 ± 3.9	<0.001
SBP, mmHg	122.9 ± 18.2	128.2 ± 18.3	134.7 ± 21.4	<0.001
DBP, mmHg	81.0 ± 10.8	84.5 ± 11.9	80.2 ± 9.1	<0.001
Current smoker, %	15.5%	13.3%	10.0%	0.4021
Statin use, %	6.2%	13.3%	10.0%	0.0011
CVD, %	16.2%	48.2%	60%	<0.001

Values are presented as no (%), mean ± SD (standard deviation); *p*-values by one-way ANOVA (continuous) or chi-square (categorical); HDL-C, high-density lipoprotein; LDL-C, Low-density lipoprotein cholesterol; HbA1c, hemoglobin A1C; SBP, systolic blood pressure; DBP, diastolic blood pressure; CVD, cardiovascular disease.

**Table 4:** Logistic regression analysis of factors associated with prevalent cardiovascular disease.

Predictor	Adjusted OR (95% CI)	*p*-Value
Possible FH	8.15 (5.30–12.53)	<0.0001
Probable FH	40.60 (9.15–180.2)	<0.0001
Age, per 1 year	1.035 (1.025–1.044)	<0.0001
Male sex	0.71 (0.52–0.90)	0.0050
Current smoker	1.04 (0.74–1.44)	0.8010
Body mass index, per 1 kg/m^2^	1.04 (1.02–1.07)	0.0010
Systolic BP, per 1 mmHg	1.005 (0.998–1.012)	0.1540
LDL-C, per 1 mmol/L	0.61 (0.52–0.71)	<0.0001

OR = odds ratio; CI = confidence interval; FH status was entered as categorical (reference = No FH). CVD includes coronary heart disease or stroke. LDL-C here is the measured value at survey (which may be lowered by treatment in some participants).

## Data Availability

The data presented in this study are available on request from the corresponding author due to institutional and national data protection regulations that restrict public sharing of health data.

## Abbreviations

The following abbreviations are used in this manuscript:

FH	Familial hypercholesterolemia
LDL-C	Low-density lipoprotein cholesterol
HDL-C	High-density lipoprotein cholesterol
TC	Total cholesterol
TG	Triglyceride
CVD	Cardiovascular diseases
ASCVD	Atherosclerotic cardiovascular disease
DLCN	Dutch Lipid Clinic Network
HbA1c	Glycated hemoglobin
BMI	Body mass index
MI	Myocardial infarction
SBP	Systolic blood pressure
DBP	Diastolic blood pressure
